# Effect of AF Surface Nanostructure on AFRP Interface Properties Under Temperature: A MD Simulation Study

**DOI:** 10.3390/polym17152024

**Published:** 2025-07-24

**Authors:** Zhaohua Zhang, Guowei Xia, Chunying Qiao, Longyin Qiao, Fei Gao, Qing Xie, Jun Xie

**Affiliations:** 1 State Key Laboratory of Alternate Electrical Power System with Renewable Energy Sources, North China Electric Power University, Beijing 102206, China; 120222101116@ncepu.edu.cn (Z.Z.); 120232101128@ncepu.edu.cn (G.X.); 220242213321@ncepu.edu.cn (C.Q.); 220222213212@ncepu.edu.cn (L.Q.); xieqing@ncepu.edu.cn (Q.X.); 2 China Electric Power Research Institute, Beijing 100192, China; feigao@epri.sgcc.com.cn

**Keywords:** aramid fiber-reinforced epoxy resin composites (AFRP), molecular simulation, interface modification, MD

## Abstract

The insulating rod of aramid fiber-reinforced epoxy resin composites (AFRP) is an important component of gas-insulated switchgear (GIS). Under complex working conditions, the high temperature caused by voltage, current, and external climate change becomes one of the important factors that aggravate the interface degradation between aramid fiber (AF) and epoxy resin (EP). In this paper, molecular dynamics (MD) simulation software is used to study the effect of temperature on the interfacial properties of AF/EP. At the same time, the mechanism of improving the interfacial properties of three nanoparticles with different properties (insulator Al_2_O_3_, semiconductor ZnO, and conductor carbon nanotube (CNT)) is explored. The results show that the increase in temperature will greatly reduce the interfacial van der Waals force, thereby reducing the interfacial binding energy between AF and EP, making the interfacial wettability worse. Furthermore, the addition of the three fillers can improve the interfacial adhesion of the composite material. Among them, Al_2_O_3_ and CNT maintain a large dipole moment at high temperature, making the van der Waals force more stable and the adhesion performance attenuation less. The Mulliken charge and energy gap of Al_2_O_3_ and ZnO decrease slightly with temperature but are still higher than AF, which is conducive to maintaining good interfacial insulation performance.

## 1. Introduction

Gas-insulated switchgear (GIS) has gradually become a core component of modern power systems due to its compact structure, small footprint, and high reliability [1]. The insulating rod is a transmission-insulating structural component that connects the operating mechanism and the body of the GIS. Its performance is directly related to the reliability and safety of power grid operation. At present, aramid fiber-reinforced epoxy resin composites (AFRP) are becoming excellent material for making insulating rods. However, the high inertness of aramid fiber (AF) leads to poor wettability with epoxy resin (EP) [2]. The formed EP/AF interface has defects and a high failure rate. Under complex working conditions, the high temperature caused by voltage and current, as well as external climate change, will further amplify this defect. Therefore, considering the degradation mechanism of the interface performance of AFRP for GIS insulating rods under the influence of different temperatures and making targeted improvements is of great significance to ensure the safe and stable operation of the power system.

Many studies have shown that nanoparticles have been widely used to modify composite materials. Nanomaterials can provide a larger interface area than macroscopic and micron-sized materials of the same volume fraction [3], which means that they can provide more contact points and achieve better modification effects. Jin et al. modified the AFRP interface by preparing carboxylate and aminated graphene oxide (CGO and AGO) and found that AGO had a better modification effect than CGO in terms of mechanical properties [4]. Dharmavarapu et al. modified AFs by silane-grafted nano-SiO_2_ and found that the tensile and flexural strengths of AFRP composites were greatly improved [5]. Xie et al. self-assembled ANFs/SiO_2_ layers onto the AF surface and found that the breakdown voltage of the composite material first increased and then decreased with the increase in the number of assembled layers [2]. Xu et al. modified the AF surface with dopamine and found that the flexural strength of the composite material was significantly improved [6]. External environmental conditions also have a great influence on the performance of insulation pull rods, and many scholars have conducted research in related fields [7,8,9]. However, current research generally focuses on mechanical stress and electric field, lacks the exploration of temperature changes, and is mostly based on macroscopic experimental levels.

As an important method for studying the microscopic properties of materials, molecular simulation has a wide range of applications in different research fields. By using molecular simulation tools, the relationship between the macroscopic properties of materials and their microscopic structures can be studied at the molecular level [10,11]. Compared with traditional experiments, molecular simulation methods have the advantages of high accuracy, low cost, and high efficiency, and the simulation results are very consistent with the experimental results [12,13]. Therefore, molecular simulation has been widely used by scholars around the world in their research, and through this method, the interface of materials can be microscopically characterized [14,15]. Lou et al. used molecular simulation methods to find that adding multi-chain crosslinking and more hydroxyl groups can enhance the interfacial bonding between silicone rubber and silica [16]. Tam et al. studied the microscopic creep behavior of the carbon fiber/EP interface under various shear load levels based on molecular dynamics (MD) [17]. According to the research of Li et al., the graphene oxide (GO)/EP interface in water has stronger interfacial van der Waals, electrostatic, and hydrogen bond interactions than the graphene (GN)/EP interface [18]. A small number of scholars have also researched the AFRP interface. Pan et al. introduced different functional groups, such as hydroxyl, carboxyl, and silane coupling agents, into the AFRP interface through MD and found that functionalization can improve the interfacial shear stress and tensile strength of polymer nanocomposites and can provide an additional mechanical interlocking effect, effectively improving the load transfer capacity [19]. Chaudhary et al. used MD simulation to study the effect of graphene on the mechanical properties of AFRP composites and found that with the increase in graphene content, the elastic modulus and ultimate strength of the system also increased monotonically [20].

Based on molecular simulation methods, this paper explores the mechanism of improving the interface performance of nano-modified AFRP under temperature changes from a microscopic perspective. The changes in system properties at the atomic level were studied from the perspectives of MD and density functional theory (DFT) using Materials Studio. Insulator Al_2_O_3_, semiconductor ZnO, and conductor carbon nanotubes (CNTs) were selected to modify AFs for comparison. The research results are of great significance for improving the AFRP interface performance of insulation pull rods and clarifying the mechanism of performance changes.

## 2. Materials and Methods

**Figure 1:**
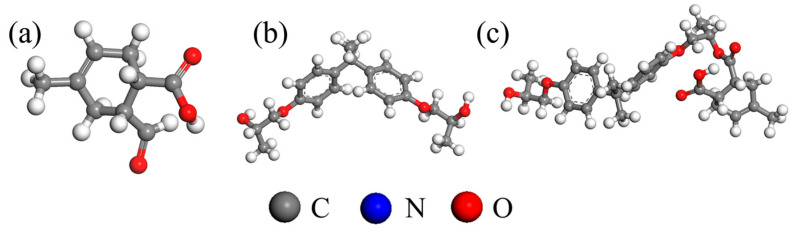
EP monomer molecule: (**a**) MTHPA; (**b**) DGEBA; (**c**) DGEBA-MTHPA.

The EP is crosslinked by methyl phthalate (MTHPA) and bisphenol A EP groups (DGEBA). Figure 1 shows the monomer model created with Materials Studio (MS). The software version number is 7.0.

The Amorphous cell module was used to fill the unit cell with 90 MTHPA and 40 DGEBA. To ensure a certain initial crosslinking degree, 10 pre-crosslinked monomer molecules (DGEBA-MTHPA) were stacked together; the initial density was set to 0.6 g/cm^3^ to ensure that the molecules had enough space for dynamic crosslinking. The unit cell parameters were α = β = γ = 90°, a = b = c = 45.33 Å. The model had a total of 4670 atoms. The EP cured product prepared using anhydride has good heat resistance and chemical stability. The main crosslinking reaction mechanism is as follows:

1. Water molecules open the EP groups at both ends of the EP to generate hydroxyl groups.
2. The anhydride group reacts with the hydroxyl group in the EP to generate an ester group and a carboxyl group.
3. The carboxyl group reacts with the EP group to generate a new ester group.
4. In an acidic environment, EP groups and hydroxyl groups undergo etherification reactions.

Step 1


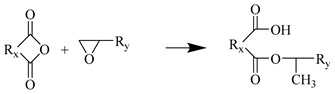


Step 2


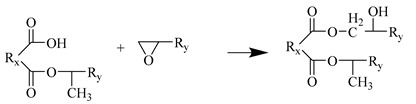


Step 3


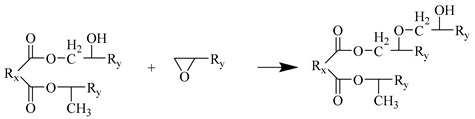


Step 4


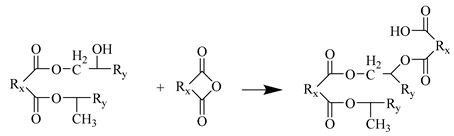


Step 5


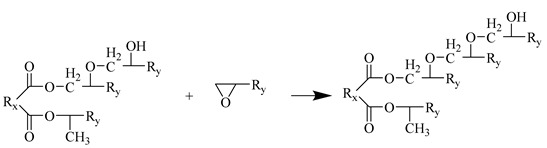


**Figure 2:**
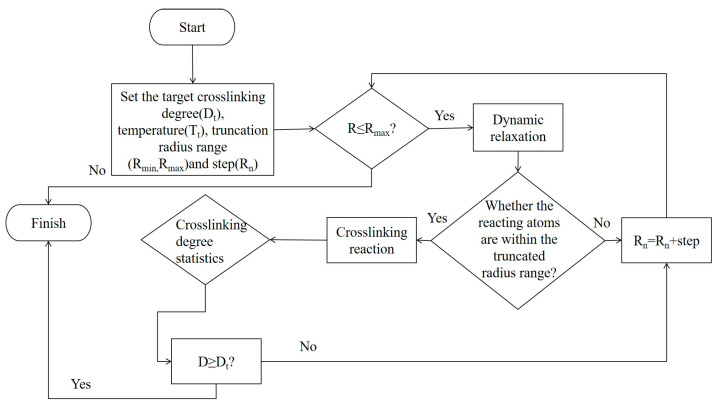
EP crosslinking script operation mechanism.

**Figure 3:**
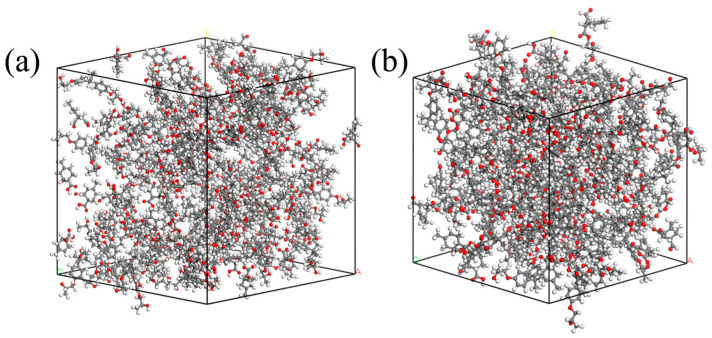
Schematic diagram of model before and after EP optimization: (**a**) initial configuration of EP; (**b**) optimized EP configuration.

A Perl script was used for crosslinking. The specific operation mechanism of the script is shown in Figure 2. The starting temperature was set to 580 K, the cutoff radius range was 3.5~10 Å, the step size was 0.5 Å, and the predetermined crosslinking degree was set to 90%. To obtain a more reasonable structure, the system needs to be optimized through methods such as geometry optimization, annealing, NVT, and NPT dynamics. This paper uses the COMPASS force field [21,22] and performs 10,000 steps of iteration based on the Smart algorithm to achieve structural optimization. The correctness of this force field has also been widely verified by many researchers [23,24,25]. The EP with optimized structure was annealed for five cycles at 298~798 K, with an annealing interval of 50 K. The unreasonable structure of the model was eliminated during the periodic heating and cooling process. The total annealing time was 1 ns. The annealed model was placed in the NPT ensemble for final optimization, with a step size of 1 fs and a cutoff radius of 15.5 Å. The van der Waals force and electrostatic interaction were calculated based on atom-based and Ewald techniques, respectively, with a simulation time of 500 ps. The model was then run in the NVT ensemble for dynamics and data collection. The Nose–Hoover Thermostat and Berendsen Barostat were used to control the temperature and pressure of the simulation. The parameters of the Nose–Hoover thermostat are set as follows: temperature difference is 10.0 K; Q ratio is 0.01; collision rate is 1.0; and decay constant is 0.1 ps. The parameters of the Berendsen Barostat are set as follows: cell time constant is 1.0 ps; and decay constant is 0.1 ps. The optimization results of the epoxy resin model are shown in Figure 3.

**Figure 4:**
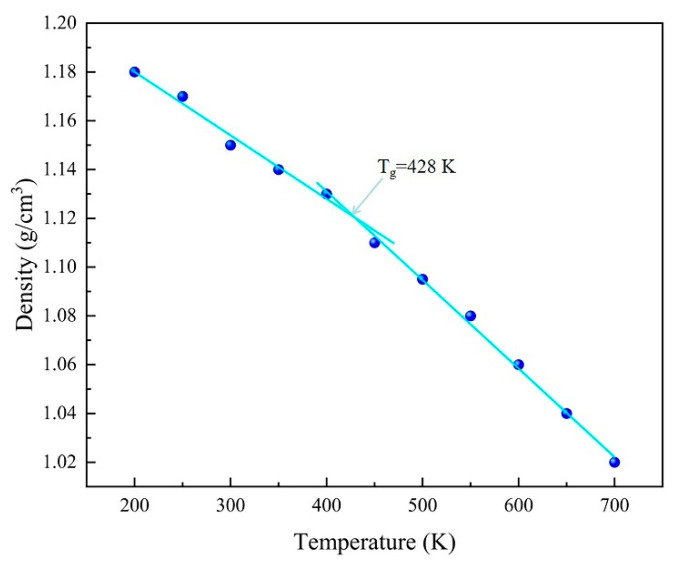
EP glass transition temperature.

One of the most important indicators of the thermal properties of a material is its glass transition temperature (Tg), which is the temperature at which a thermosetting resin changes from a glassy state to a highly elastic state. The glass transition temperature of the EP model was determined using a Perl script, and an NPT ensemble dynamics study based on the COMPASS force field was performed in the range of 200 K to 700 K with a temperature interval of 50 K. The glass transition temperature of the EP is 428 K, as shown in Figure 4, which is consistent with the experimental data (436 K) [26], thus proving the reliability of the model.

**Figure 5:**
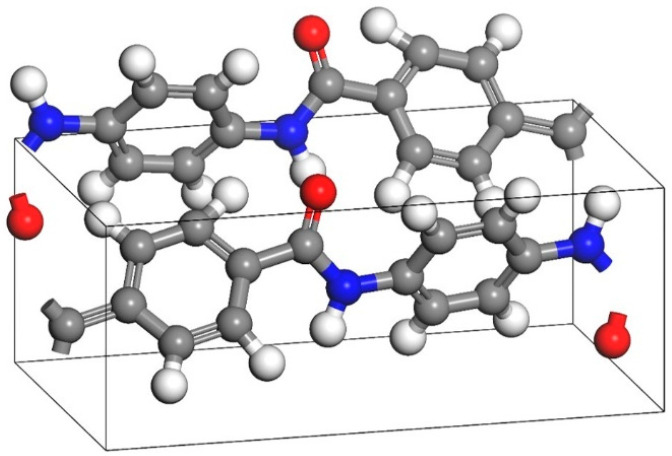
Original AF model.

**Figure 6:**
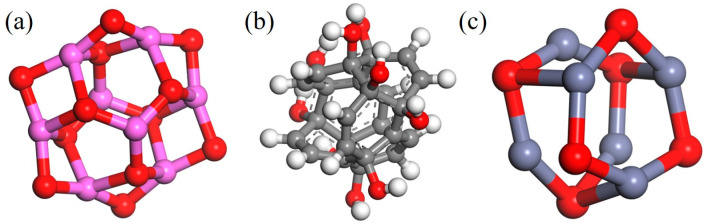
Three types of nanoparticles: (**a**) Al_2_O_3_; (**b**) CNT; (**c**)ZnO.

The AF unit cell structure (1176049) was obtained from the Cambridge Crystal Data Center (CCDC), as shown in Figure 5. The monomer models of the three types of nanoparticles are shown in Figure 6. Some scholars have verified the reliability of this model by comparing it with experiments [1]. Energy minimization and MD optimization were performed based on the COMPASS force field. The optimized model was supercelled so that the size of the AF unit cell could match that of the EP. The EP-AF interface model was established by building layers, and the vacuum layer thickness was set to 40 Å to eliminate the influence of the periodic structure. The interface model was optimized according to the EP optimization method until equilibrium was achieved to achieve a more stable and reasonable configuration.

**Figure 7:**
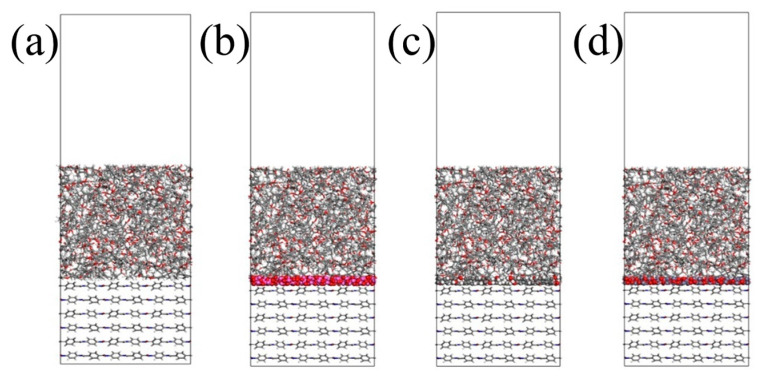
Interface dynamics model: (**a**) original AFRP interface (AF/EP); (**b**) Al_2_O_3_-modified interface (AF/Al_2_O_3_/EP); (**c**) CNT-modified interface (AF/CNT/EP); (**d**) ZnO-modified interface (AF/ZnO/EP).

A chiral (3,3) CNT with a diameter of 4.07 Å and a length of 4.92 Å was constructed and hydroxylated. On the one hand, the polar groups are used to strengthen the connection strength of the interface. On the other hand, since the conductivity of a CNT is much stronger than that of general conductors, the Fermi level barrier between the polar groups and the CNT weakens the electron transfer performance of the CNT to a certain extent, reducing its adverse effect on the insulation performance of the composite system. Al_2_O_3_ and ZnO clusters with a radius of 3.4 Å were constructed to ensure that the three nanoparticles have similar relative molecular masses. The AF was modified by establishing a good cluster model, and then the interface model was built and optimized with EP. To eliminate the periodic effect, a 40 Å vacuum layer was added above the interface. The kinetic models of the four structures are shown in Figure 7.

Some scholars have calculated that the maximum temperature rise in the internal environment of GIS under different working conditions is about 70~80 °C [27]. With a certain margin, the simulation temperature is set to 298~378 K, and the temperature interval is 20 K. This paper uses COMPASS force field simulation throughout, and the step size is set to 1 fs.

## 3. Results and Discussion

### 3.1. Interface Adhesion Ability

#### 3.1.1. Interface Binding Energy

**Figure 8:**
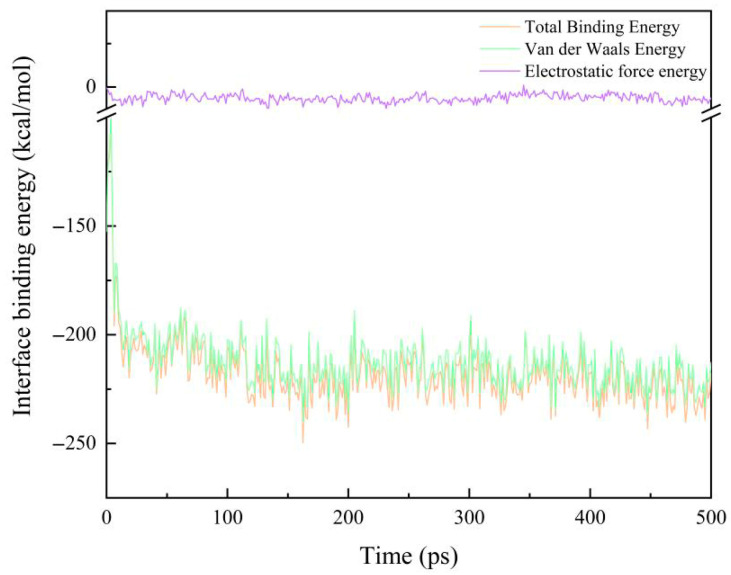
A schematic diagram of the change in the original AF/EP interface binding energy at 298 K.

Interface binding energy refers to the energy released when atoms or other particles bind to each other to make the system more stable. It is a parameter that characterizes the interaction energy of different components in a mixed system and can directly measure the bonding strength between the EP and AF interface. The binding energy is calculated for each interface model. The calculation formula for the binding energy is as follows:

(1) $$E_{int}=E_{total}-E_{EP}-E_{AF}$$

where *E*_total_ is the total energy of the system, *E*_EP_ is the energy of the EP, and *E*_AF_ is the energy of the modified AF system. The reliability of the calculation results is ensured by calculating the average value of the last 30 frames of the trajectory file. As shown in Figure 8, it is a schematic diagram of the change in the binding energy of the original interface model at room temperature (298 K) with the simulation time. It can be seen that with the extension of the simulation time, the binding energy between the interfaces eventually tends to be stable.

**Figure 9:**
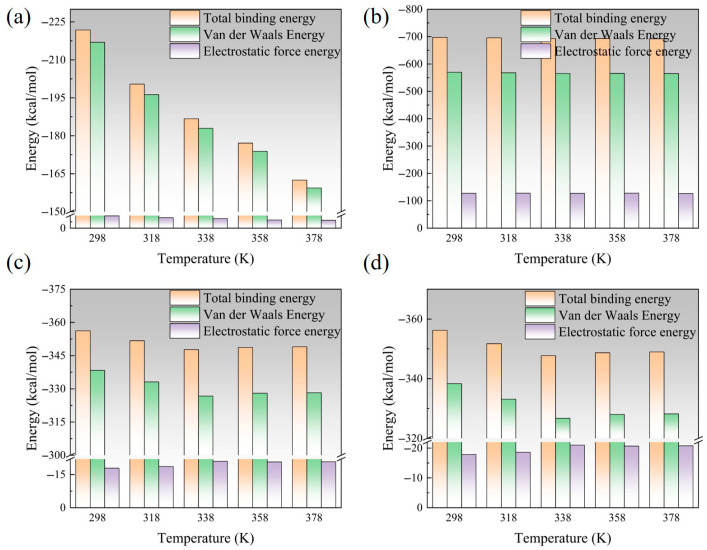
A schematic diagram of the change in binding energy of each interface at different temperatures: (**a**) EP/AF; (**b**) EP/Al_2_O_3_/AF; (**c**) EP/CNT/AF; (**d**) EP/ZnO/AF.

Figure 9 is a schematic diagram of the change in interface binding energy at different temperatures. With the increase in temperature, the interface binding energy shows a certain degree of decrease. After nano-modification, the interface binding energy is greatly improved, and the overall effect is EP/Al_2_O_3_/AF > EP/CNT/AF > EP/ZnO/AF > EP/AF. After Al_2_O_3_ modification, the interface binding energy can be increased by more than 200%, from 221.81 kcal/mol of the original interface at room temperature to 697.33 kcal/mol. It is much higher than the other two modification methods. After CNT modification, the increase is about 50%, and the zinc oxide modification is less than 50%. Although the temperature rise will also lead to a decrease in the adhesion ability of the modified interface, it is still higher than the original AFRP interface at the same temperature. Among them, the interface binding energy after Al_2_O_3_ and CNT modification changes less with temperature, and the difference at different temperatures is within 10 kcal/mol. The interface binding energy after the original interface and ZnO modification changes greatly, in the range of 40 kcal/mol~50 kcal/mol.

Binding energy can be divided into two parts: electrostatic energy and van der Waals energy. To further explore the changing law of interface binding energy under different conditions, schematic diagrams of interface electrostatic energy and van der Waals energy under different conditions are also given. In the four cases, the interface binding energy is mainly van der Waals energy. The original AFRP interface has almost no electrostatic energy (below 5 kcal/mol), the electrostatic energy is slightly improved after CNT and ZnO modification, and the interface electrostatic energy is greatly improved after alumina modification (above 100 kcal/mol). This may be due to the large surface electrostatic potential of Al_2_O_3_. The electrostatic energy of the original AFRP interface and the interface after ZnO modification does not change much with temperature, and the change in van der Waals energy is the main reason for the change in its binding energy. For the interface modified by Al_2_O_3_ and CNT, their van der Waals energy and electrostatic energy are relatively stable. Van der Waals forces are divided into dispersion forces, orientation forces, induction forces, etc., all of which are related to the dipole moment of the molecule [28,29]. This may be because Al_2_O_3_ and CNT have larger dipole moments and their dipole moments can maintain a relatively stable value with temperature changes, so their binding energy will maintain a relatively high and stable value.

#### 3.1.2. Wettability

**Figure 10:**
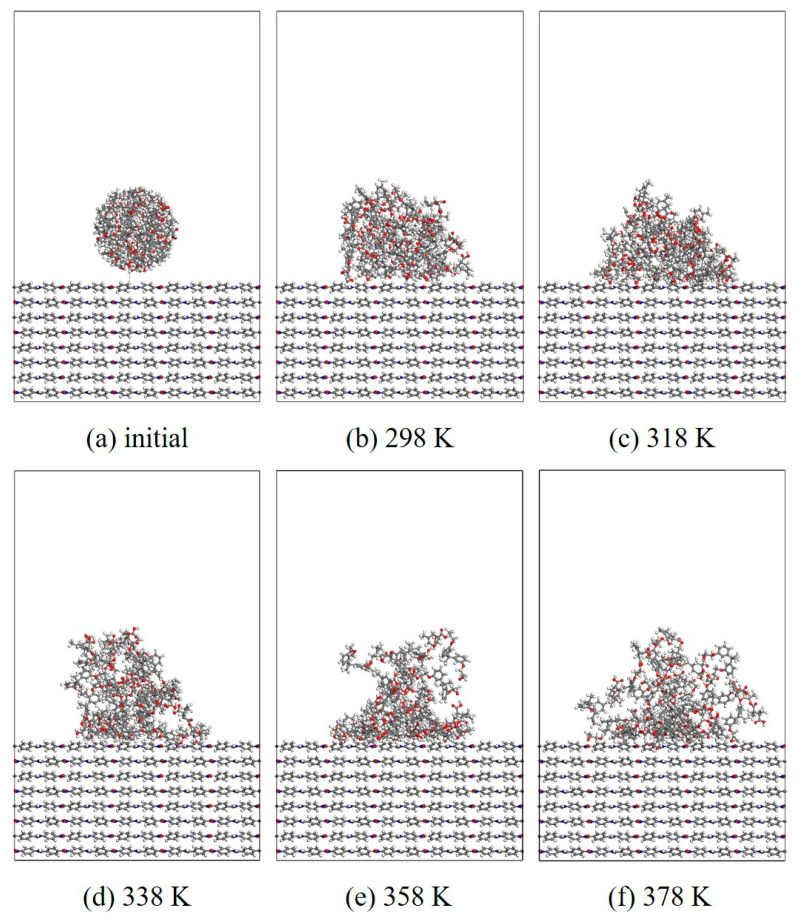
Evolution of wettability of EP/AF at different temperatures.

**Figure 11:**
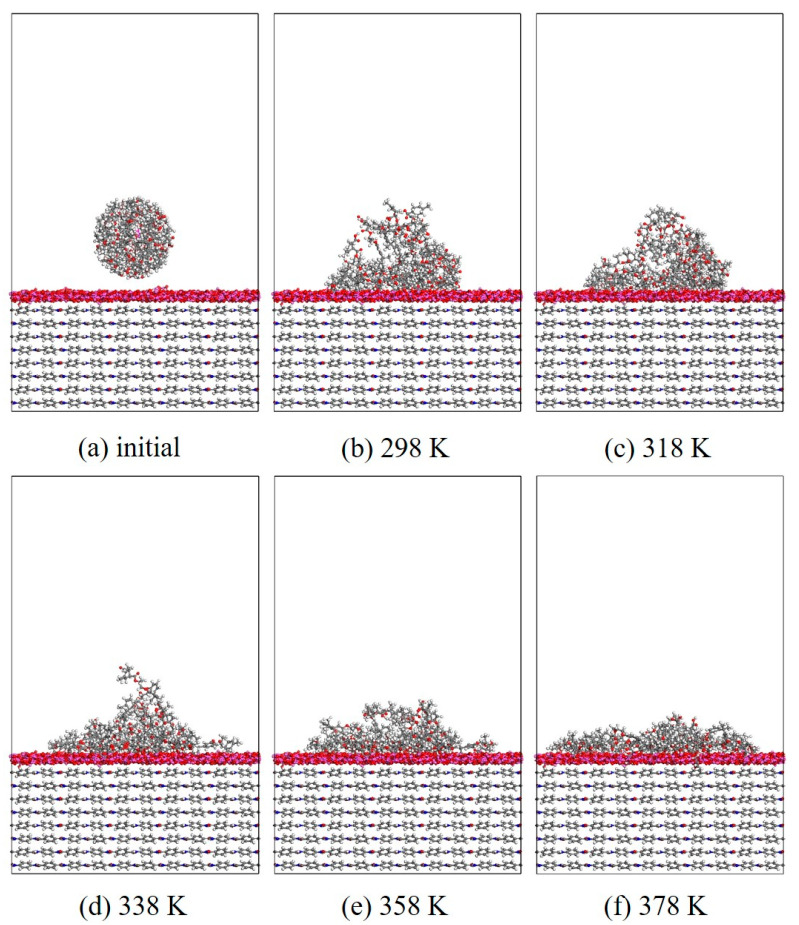
Wettability evolution diagram of EP/Al_2_O_3_/AF at different temperatures.

**Figure 12:**
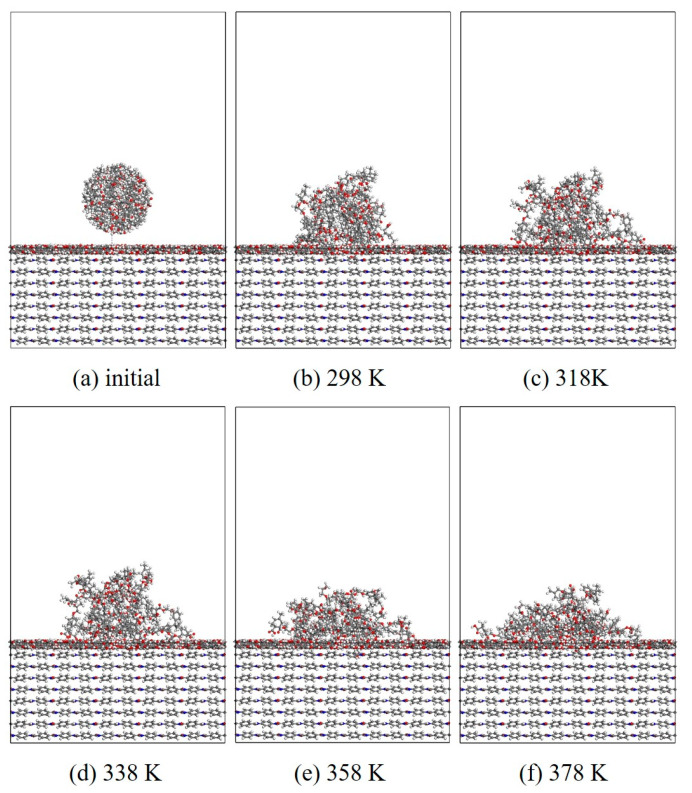
Evolution of wettability of EP/CNT/AF at different temperatures.

**Figure 13:**
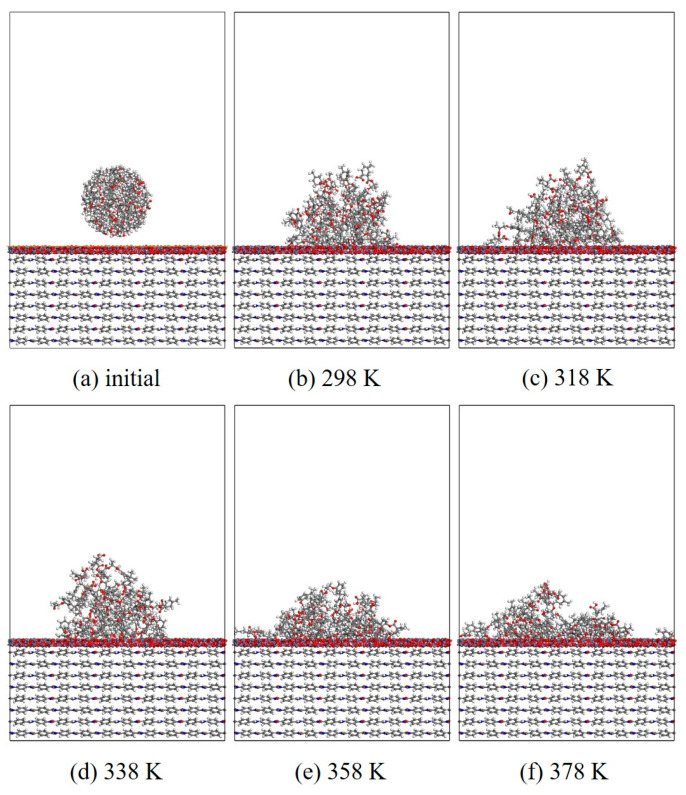
Evolution of wettability of EP/ZnO/AF at different temperatures.

In this section, MD simulation is used to study the EP wetting behavior on the AF surface. The wettability is determined by the balance between the interfacial binding energy and the intermolecular forces within the EP. A new AF unit cell model with a substrate thickness of 30 Å and a modified material thickness of 3 Å is created. A new EP cluster model with a radius of 15 Å is created through the optimized EP, ensuring that the center of mass of the EP cluster is 14 Å away from the upper surface of the interface model, that is, the bottom of the cluster is about 2 Å away from the upper surface of the interface. Considering the size of the cluster itself, a new vacuum layer with a thickness of 70 Å is created above the interface model. The model is run in the NVT ensemble for 500 ps, and the Andersen thermostat is used to control the temperature. The van der Waals force and electrostatic interaction are calculated based on the atom-based and Ewald techniques. During the wettability simulation, the model will show a series of dynamic changes and finally form the corresponding trajectory file. The 1000th frame of each trajectory file is taken for comparison. Figure 10, Figure 11, Figure 12 and Figure 13 are schematic diagrams of the comparison of interface wettability before and after modification at 298–378 K. As the temperature increases, the contact area between the EP clusters and the modified interface gradually increases, which is undoubtedly a manifestation of enhanced wettability between the two. This may be due to the increase in temperature, where the molecular thermal motion is enhanced, and the interaction force between EP molecules is reduced, making it easier for the EP to spread on the surface. However, for the original AFRP system, the increase in temperature also leads to more “gaps” in the EP above the interface, which means that the free volume of the EP increases. On the one hand, it may be due to the weak adsorption of the original AF interface on the EP. On the other hand, it may be due to the increase in temperature. The enhanced atomic mobility is more likely to show a tendency to break free from the interface constraints. The increase in free volume will lead to a weakening of the overall wettability of the EP and the interface, thereby reducing the interface adhesion ability to a certain extent, although the strength of the interface bonding is also closely related to the charge distribution of the atoms. At the same time, these “gaps” will provide a lot of space for the transmission of electrons, which will reduce the insulation performance of the interface to a certain extent.

#### 3.1.3. Evolution of Interfacial Free Volume Under Stress State

**Figure 14:**
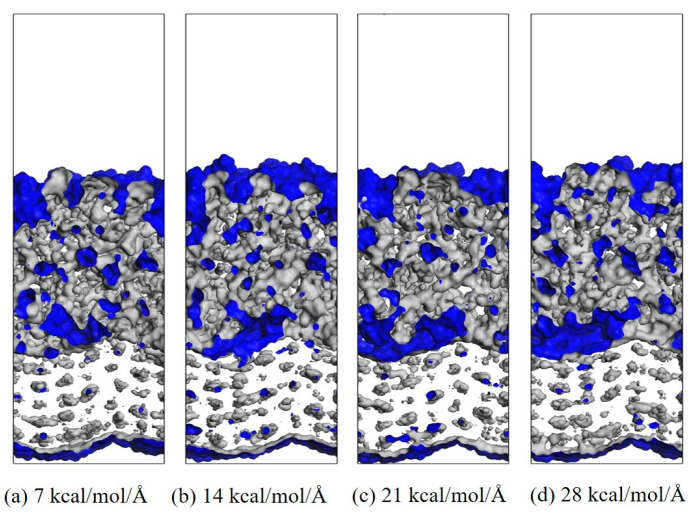
Example of system free volume (EP/AF at 298 K).

**Figure 15:**
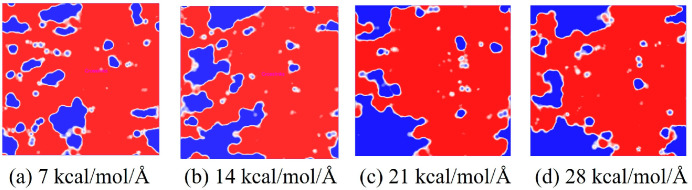
Free volume slice.

**Figure 16:**
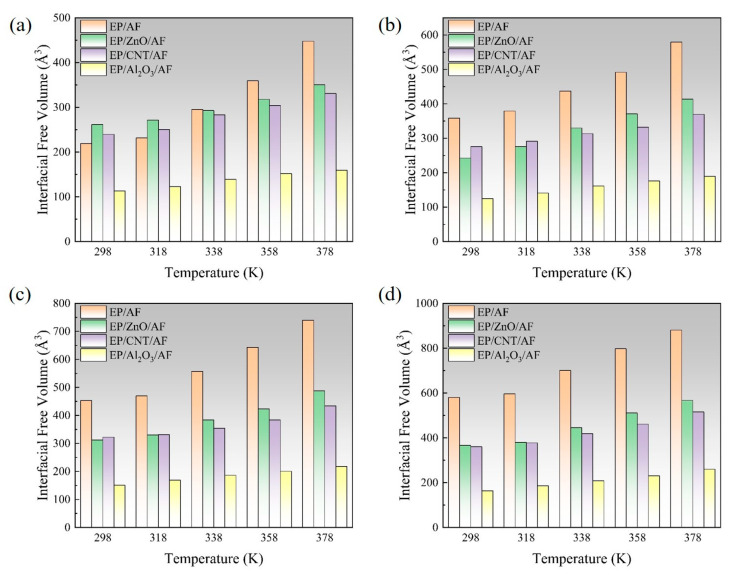
Schematic diagram of free volume of each interface changing with temperature and tension: (**a**) 7 kcal/mol/Å; (**b**) 14 kcal/mol/Å; (**c**) 21 kcal/mol/Å; (**d**) 28 kcal/mol/Å.

In the process of transmitting the action of the operating mechanism in GIS to the main unit, the insulation pull rod needs to withstand various mechanical loads [30,31]. According to the research of relevant scholars, the maximum tension that the insulation pull rod bears when it moves is about 130 MPa [32]. The length and width of the interface model are about 37.6 × 36.4 Å^2^, so the tension to be given should be between 0 and 25.6 kcal/mol/Å. Considering that a certain margin should be left, the tension given to the model in this calculation is between 0 and 28 kcal/mol/Å. The corresponding tension is applied to the modified AF through a Perl script, and the change in the free volume of the interface after the tension is applied is calculated to measure the damage to the interface caused by different tensions. The internal volume of the polymer is divided into two parts: free volume, that is, the unoccupied part of the polymer, and occupied volume, that is, the intrinsic volume occupied by the molecule. A spherical probe with a radius of 1.0 Å is used to move on the van der Waals surface to generate the Connolly surface, and the volume between it and the probe atom is the free volume. The interface free volume is calculated using Formula (2). Where *FV*_all_ is the total free volume, *FV*_EP_ is the EP free volume, and *FV*_AF_ is the free volume of the modified AF system. As shown in Figure 14, taking the original AFRP interface as an example, the interface free volume change diagram corresponds to the model at room temperature (298 K) under 7 kcal/mol/Å, 14 kcal/mol/Å, 21 kcal/mol/Å, and 28 kcal/mol/Å tension, where the occupied volume and free volume are represented by gray and blue, respectively, and the white area is the vacuum area. In the figure, as the applied tension increases, the interface free volume becomes larger and larger. To observe the changes at the interface more clearly, the corresponding free volume is cut, and the blue area is the free volume, as shown in Figure 15. The change in interface free volume under different tensions and temperatures is calculated, and the results are shown in Figure 16. At the same temperature, as the interface tension increases, the interface free volume expands, which is consistent with the actual situation. The expansion of the interface free volume means that the interface bonding performance deteriorates, and it also provides more space for electron transmission, making the system more susceptible to breakdown. It should be noted that although the original interface’s free volume is even smaller than that after modification under certain temperatures and certain stresses, this does not mean that the original interface has better bonding performance or electrical properties because the interface’s electrical and mechanical properties are also related to the material of the interface itself and the amount of charge carried. With the increase in temperature or external force, the damage to each interface tends to be serious, and the change in interface free volume accelerates. When the tension is 28 kcal/mol/Å, the original interface free volume expands by 301 Å^3^ during the temperature rise from 298 K to 378 K. The free volume of Al_2_O_3_ after modification changes slightly, expanding by about 96 Å^3^ under such extreme conditions. The free volume changes in ZnO and CNT after modification are moderate, with corresponding values of 200 Å^3^ and 155 Å^3^, respectively. It can be seen that under different external forces, the Al_2_O_3_-modified interface is least affected, which is consistent with its excellent static binding energy in the previous section. On the one hand, this means that it is not easily damaged by temperature and external forces during the dynamic process, resulting in interface adhesion failure, and on the other hand, it will make interface breakdown more difficult.

(2) $${FV}_{interface}={FV}_{EP}+{FV}_{AF}-{FV}_{all}$$

### 3.2. Interface Thermal Conductivity

**Figure 17:**
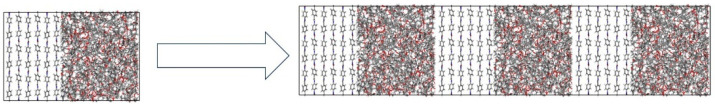
Model supercell.

**Figure 18:**
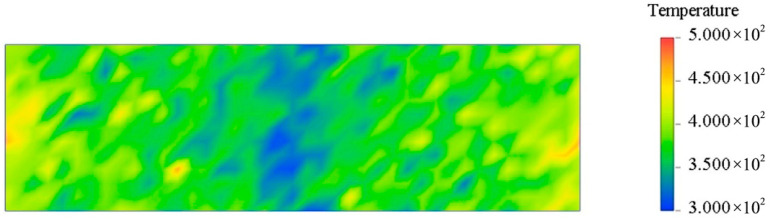
Temperature gradient visualization distribution.

**Figure 19:**
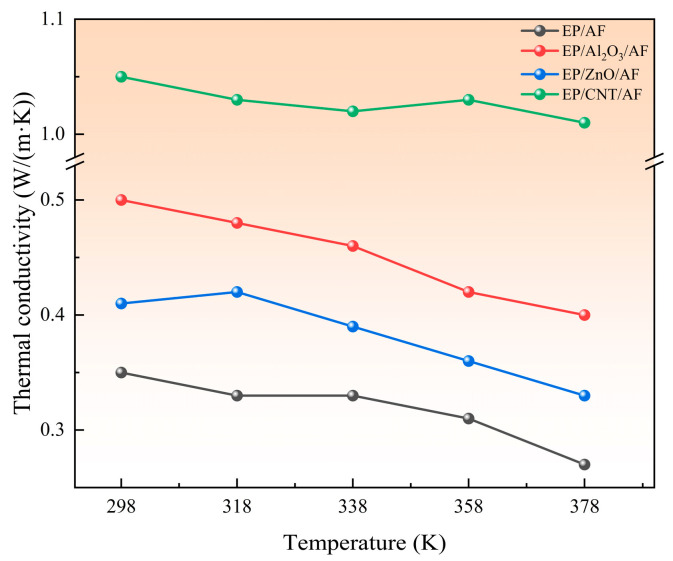
Thermal conductivity distribution of each system at different temperatures.

The two basic methods for simulating the thermal conductivity of a system by MD are equilibrium MD (EMD) and nonequilibrium MD (NEMD). EMD uses the GREEN-KUBO formula to obtain thermal conductivity. NEMD calculates thermal conductivity by defining hot and cold regions according to Fourier’s law. The EMD method requires the calculation of thermal conductivity in all directions. In contrast, the NEMD method only needs to calculate thermal conductivity in one specific direction [33]. This section uses the NEMD method to calculate thermal conductivity. NEMD can maintain steady-state flux by simply exchanging particle momentum and ensuring the conservation of total energy and total linear momentum [34,35]. In two fixed regions, the fastest atomic speed in one region is replaced by the slowest atomic speed in the other region. As a result, the temperature of the first region decreases and the temperature of the second region increases, and energy flows from region one to region two. The system is then subjected to an energy flow in the opposite direction. Eventually, when the exchanged energy cancels the return energy, a temperature gradient is formed between the two regions, and a steady state is reached. After the system reaches a steady state, the thermal conductivity is calculated according to Fourier’s law. The specific formulas are shown in Equations (3) and (4). Where (∆E) represents the energy difference, ∆t represents the time interval, A represents the cross-sectional area in the heat flow direction, and J represents the heat flux density. The NEMD method evenly cuts the model into several layers along a certain direction. This calculation is divided into 40 layers. To ensure the reliability of the results, it is necessary to ensure that the unit cell length in the heat flow direction is long and that the Z direction is processed accordingly, as shown in Figure 17. Set the time step to 1 fs, solve the corresponding thermal conductivity at different temperatures, and finally form a stable temperature gradient, as shown in Figure 18, which means the solution is complete. Figure 19 shows the thermal conductivity of different interface systems. It can be seen that the order of the thermal conductivity of the four interfaces at the same temperature is EP/CNT/AF > EP/Al_2_O_3_/AF > EP/ZnO/AF > EP/AF. After CNT modification, the interface thermal conductivity can be increased to about 3 times the original interface thermal conductivity. This is because the CNT’s thermal conductivity is excellent, far better than AF. After doping, the thermal conductivity of the composite system can be greatly improved. The covalent functional groups grafted by CNTs enhance the interaction between the doped particles and the matrix, making the molecular chains more closely bonded, and increasing the mean free path of electron and phonon transmission, thereby significantly improving the thermal conductivity of the system. The thermal conductivities of the four systems all show a decreasing trend as the temperature increases. This is because when the temperature increases, the thermal motion speed of electrons and phonons increases, and the collision with the lattice points is frequent, the mean free path is shortened, and the thermal conductivity decreases. Relatively speaking, the thermal conductivity decreases more significantly after Al_2_O_3_ modification, and the thermal conductivity decreases by 0.1 W/(m·K) when the temperature increases from 298 K to 378 K. The original interface and the ZnO-modified interface are followed by 0.08 W/(m·K). The CNT-modified interface has the smallest change, only 0.04 W/(m·K). This shows that the temperature change has a greater impact on the interfacial thermal resistance of the Al_2_O_3_-modified system. Temperature mainly affects the interfacial phonon transmission, while insulators such as Al_2_O_3_ have almost no free electrons for heat transmission, and their thermal conductivity depends entirely on phonon transmission. Therefore, the interfacial thermal conductivity is most damaged at high temperatures after adding Al_2_O_3_ nanoparticles.

(3) $$J=\frac{1}{2A}\frac{\bar{∆E}}{∆t}$$

(4) TC=1dTdz

### 3.3. Interface Electrical Properties

#### 3.3.1. Electrostatic Potential and Mulliken Charge

**Figure 20:**
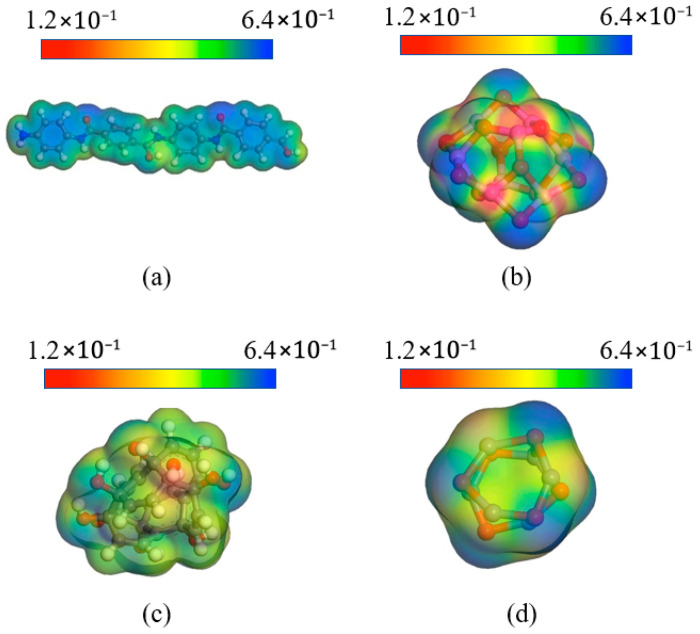
Electrostatic potential distribution of different nanoparticles: (**a**) AF; (**b**) Al_2_O_3_; (**c**) CNT; (**d**) ZnO.

The electrostatic potential and Mulliken charge of nanoparticles and AFs were determined to further understand the electrical properties of the composite system. The simulation was performed by DMol3, using the generalized gradient approximation (GGA) and the Perdew–Burke–Enzerhof functional (PBE). The pseudopotential was set to DFT Semi-core Pseudopots, which takes into account both accuracy and calculation speed. The cutoff radius was 5.5 Å, and the calculation accuracy was fine. After optimizing the geometry of each molecule, different temperatures were set in the NVT ensemble for the 300 fs dynamic simulation. Figure 20 shows the results of the DFT calculation. It can be seen that the positive and negative electrostatic potential values of Al_2_O_3_ filler are relatively high. Higher electrostatic potential can improve the compatibility of the system and increase the interaction force with surrounding molecules, thereby obtaining a modified composite system with a smaller interfacial free volume. Relatively speaking, although the electrostatic potential of CNT and ZnO is better than that of AF, it is obvious that the potential value is smaller than that of Al_2_O_3_, so its mechanical properties are relatively weak. Electrostatic potential also has a significant impact on electrical properties. Higher electrostatic potential has a stronger attraction for electrons, which is beneficial to improving the insulation capacity of the material.

**Figure 21:**
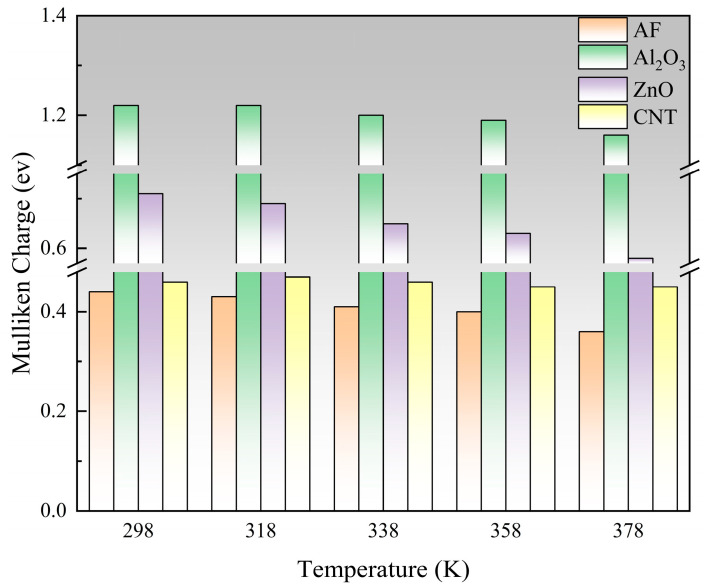
Changes in Mulliken charge of different nanoparticles under influence of temperature.

In a molecule, a positively charged atom can be considered as an electron trap, which attracts and captures neighboring free electrons or other negative charges [36,37,38]. The electron cloud tends to transfer to atoms with greater electronegativity, and different Mulliken charges can be generated due to the different electronegativity of the connected atoms [39]. A larger Mulliken charge means a larger trap energy level, which helps the system have a higher breakdown strength. To objectively characterize the charge distribution of various nanoparticles, their maximum Mulliken positive charge was calculated. The statistical results are shown in Figure 21, and the ranking is Al_2_O_3_ > ZnO > CNT > AF. Al_2_O_3_ has the largest Mulliken positive charge, which is significantly higher than the other three materials, indicating that Al_2_O_3_ can provide a more effective trap to capture electrons, which is conducive to the system having a higher breakdown strength. Although the Mulliken charge of ZnO particles is slightly smaller, it still has a significant advantage over AF. The maximum Mulliken charge of the CNT is quite close to the AF, indicating that even if a large number of polar groups are added, it is difficult to construct an effective charge trap. As the temperature rises, the Mulliken charge of ZnO particles decreases most severely, while CNTs have almost no significant fluctuations. This may be because for semiconductors like ZnO, the carrier concentration increases significantly with increasing temperature, which can enhance the charge polarization of atoms, thus causing significant fluctuations in the Mulliken charge. However, the electrons inside CNTs are highly delocalized, and the Mulliken charge is relatively small, so temperature is unlikely to have a significant effect on them.

#### 3.3.2. Molecular Orbitals

**Figure 22:**
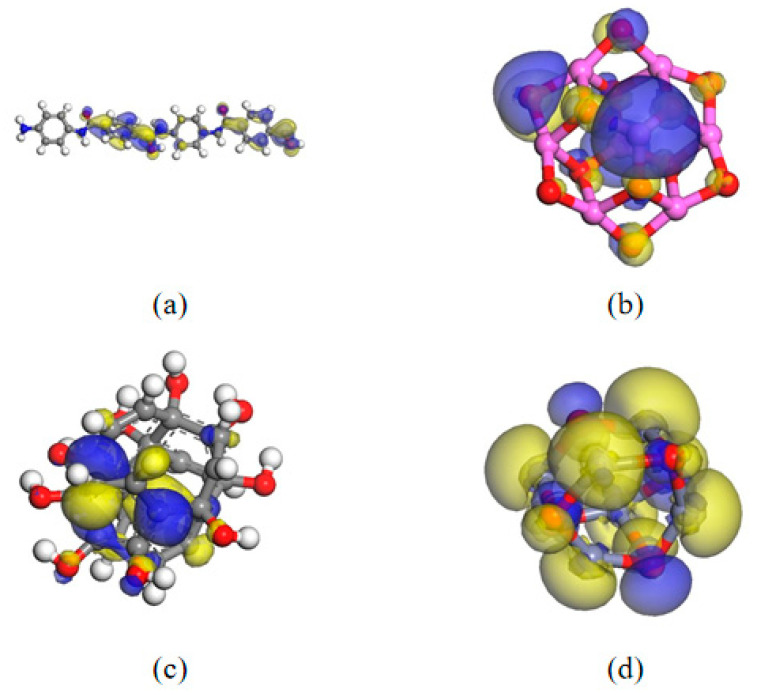
Four material orbital distributions: (**a**) AF; (**b**) Al_2_O_3_; (**c**) CNT; (**d**) ZnO.

This section studies the HOMO/LUMO distribution of AFs and various nanoparticles. DFT calculations were performed using GGA and PBE functionals through DMol3. The HOMO energy level is the orbital with the highest energy occupied among all molecular orbitals. It is easy to lose electrons. The larger the value, the easier it is to lose electrons. The LUMO energy level is the lowest unoccupied molecular orbital. It is easy to be approached by electrons. The smaller its value, the easier it is to accept electrons. In addition, the energy gap (G), that is, the energy difference in HOMO/LUMO, can be used to determine the difficulty of electron migration, and thus, to a certain extent, can reflect the breakdown characteristics. Figure 22 is the HOMO/LUMO distribution of AF and three nanomaterials at 298 K.

**Figure 23:**
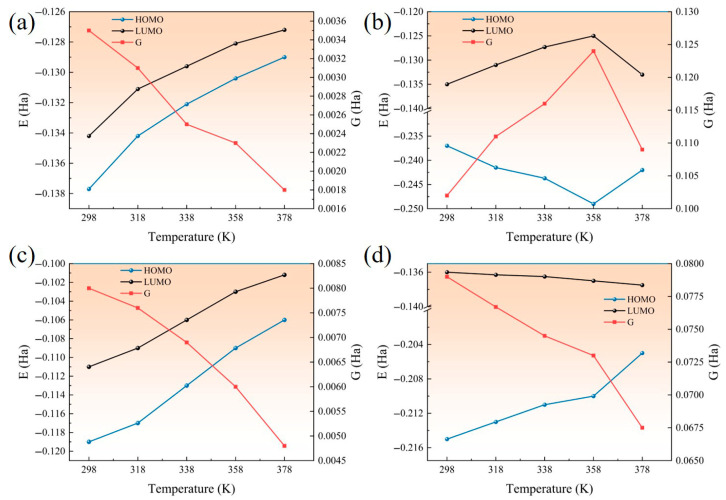
Changes in molecular orbitals of four materials with temperature: (**a**) AF; (**b**) Al_2_O_3_; (**c**) CNT; (**d**) ZnO.

The distribution of HOMO/LUMO and energy gap G of Al_2_O_3_, ZnO, CNT, and AF at different temperatures is shown in Figure 23. The order of energy gap G is Al_2_O_3_ > ZnO > AF > CNT, indicating that the addition of Al_2_O_3_ and ZnO can play a positive role in the insulation performance of the AFRP interface, while the addition of CNT will have an adverse effect. For AF and CNT, HOMO and LUMO both increase with increasing temperature, which can be explained by the following two aspects: First, the increase in temperature leads to an increase in the distance between material molecules, weakening the interaction between electrons, so that the HOMO and LUMO energy levels rise at the same time. Secondly, the increase in temperature enhances the electron–phonon interaction, which may lead to an overall upward shift in the energy level. On the one hand, the increase in HOMO and LUMO will make HOMO more likely to lose electrons, and on the other hand, LUMO will not easily obtain electrons. It can be found from the energy gap G that it is generally decreasing. It shows that considering the changes in HOMO/LUMO, the interface as a whole will become easier to break down. For ZnO, HOMO increases with increasing temperature, while LUMO decreases with increasing temperature. HOMO is more likely to lose electrons, LUMO is more likely to gain electrons, and the interface as a whole is more likely to break down. The orbital changes of Al_2_O_3_ at different temperatures are more complicated. Before 358 K, HOMO and LUMO show a decreasing and increasing trend, respectively: G increases and the interface breakdown becomes more difficult. After 358 K, the trend is the opposite, and the interface is more likely to break down. Overall, the energy gap G of Al_2_O_3_ nanoparticles has not decreased significantly, which indicates that the temperature increase will not excessively damage the insulation properties of the corresponding interface.

## 4. Conclusions

This research used molecular simulation to study the effect of AF surface nanostructure on AFRP interface performance under temperature by calculating the changes in adhesion, thermal conductivity, and electrical properties. The specific conclusions are as follows:

An increase in temperature will weaken the van der Waals force at the AFRP interface and reduce the interfacial adhesion. This will lead to the formation of a large number of voids in the EP and the deterioration of the interfacial wettability. At the same time, high temperatures will also reduce the interfacial thermal conductivity, forming a vicious cycle and aggravating the impact of high temperature on interfacial performance. In addition, the reduction in the Mulliken charge of the AF molecule (AF) and the narrowing of the orbital energy gap will lead to a decrease in interfacial insulation performance.

The introduction of three types of nanoparticles makes the interface adhesion better than the original interface at high temperatures. For the interfacial adhesion ability, Al_2_O_3_ and CNT nanoparticles maintain a high, stable dipole moment at high temperatures, giving the interface a strong and stable van der Waals force energy so that its high-temperature adhesion remains excellent. Although high temperature weakens the interfacial binding energy, the enhanced molecular motion has become the dominant factor in the improvement of interfacial wettability. In terms of interfacial thermal conductivity, although nano-modification cannot completely suppress the attenuation of high-temperature thermal conductivity, effective heat transfer can still be maintained within the operating temperature range, which can effectively avoid further temperature increases, thereby alleviating damage to the interface performance. In terms of interfacial electrical properties, the Mulliken charge and energy gap of Al_2_O_3_ and ZnO particles at high temperatures are greater than AF, indicating that the insulation performance of the modified interface is better and that the material can still have excellent insulation properties at high temperatures.

## Data Availability

The original contributions presented in this study are included in the article. Further inquiries can be directed to the corresponding author.
